# Obstacles and facilitators to communicating with children about their parents’ mental illness: a qualitative study in a sub-district of Mpumalanga, South Africa

**DOI:** 10.1186/s12888-023-04569-3

**Published:** 2023-01-27

**Authors:** Lucy Dean, Hadassah Buechner, Bianca Moffett, Meriam Maritze, Louise J. Dalton, Jeffrey R. Hanna, Elizabeth Rapa, Alan Stein, Stephen Tollman, Kathleen Kahn

**Affiliations:** 1 Department of Psychiatry, University of Oxford, Oxford, UK; 2 MRC/Wits Rural Public Health and Health Transitions Research Unit (Agincourt), School of Public Health, University of the Witwatersrand, Johannesburg, South Africa; 3 School of Nursing and Midwifery, Queen’s University Belfast, Belfast, UK

**Keywords:** Communication, Children, Parental depression, Healthcare professionals

## Abstract

**Background:**

Given that common mental disorders are one of the leading causes of disease burden worldwide, it is likely that many children are growing up with a parent or other adult within their family who has anxiety or depression. Parents with a mental illness may not consider it appropriate to discuss their illness with their child, and consequently an absence of communication may lead to stigmatization, shame, misunderstanding their parents’ symptoms, and even blaming themselves. There is a scarcity of research exploring the experiences and perceptions of healthcare professionals about communication with children of parents with mental illness in low-resource and African contexts.

**Methods:**

A qualitative study using semi-structured interviews with healthcare professionals (*n* = 15) was conducted within the Bushbuckridge sub-district of Mpumalanga Province, South Africa. Data were analysed using Thematic Analysis.

**Results:**

Four themes were identified relating to the obstacles around communication with children. These included: (1) finding an appropriate language to describe mental illness, as well as the prevailing cultural explanations of mental illness (2) the stigma associated with mental illness (3) the perceived role of children in society and (4) mental health services and staff skills. Two themes that addressed facilitators of communication about parental mental illness were identified: (1) the potential to increase mental health awareness amongst the broader community through social media, the internet, and general psychoeducation (2) healthcare professionals’ concerns for the wellbeing and future mental health of patients’ children, as well as their hopes for increased mental health awareness amongst future generations.

**Conclusions:**

This study provides insight into healthcare professionals’ attitudes and perceptions about talking to patients and families within their community about mental illness. The results provide recommendations about possible ways to promote sharing information about a parent’s mental illness with children at an individual and community level. Future research should focus on the collaborative creation of culturally sensitive psychoeducational resources and evidence-based guidelines. This must be supported by systemic and organisational change in order for professionals to successfully facilitate conversations with patients who are parents, and their children.

## Background

Common mental illnesses are one of the leading causes of disease burden worldwide, and account for 19% of all years lived with disability [1]. In South Africa, approximately 16.5% of the population will experience a common mental illness (including anxiety and depression) in a 12-month period [2, 3], and a third of the population will experience a mental illness in their lifetime [4]. Importantly, these estimates are higher than the international prevalence for mental illness [5]. Furthermore, South Africa has a large mental health treatment gap, with an estimated 75% of people with mental illness unable to access treatment of any kind [6]. Several factors contribute to this, not least that mental health budgets constitute a small fraction of total health spending in South Africa [7, 8]. In addition to financial constraints, a review of barriers to the improvement of mental health services in low- and middle-income countries (LMICs) highlighted the limited number of trained professionals and centralisation of mental health resources to urban areas [9].

Many people who experience a mental illness are also parents of children (< 18 years old), with a recent systematic review reporting a prevalence of parents in adult psychiatric services ranging from 12.2 to 45%, although it is important to note these studies were all conducted in high income countries [10]. Prior research has shown that children of parents with a mental illness have a higher risk of adverse social, developmental and psychological outcomes, as well as experiencing a poorer relationship with their parents [11–13]. Parents with mental illness may not consider it appropriate to discuss their illness with their child [14], fear that it will be ‘burdensome’ for their child, or may think that their child will not understand their illness and its manifestations [15]. However, without an explanation children may struggle to understand their parent’s symptoms [16]. Consequently, children’s perception of parental mental illness will be based solely upon their observations and what they have heard from other adults in their network which may perpetuate worries and misconceptions [17]. Children’s understanding of illness also varies across different ages. It is especially important to acknowledge the concept of ‘magical thinking’ where a child may believe that their thoughts or actions have caused their parents’ illness [18]. Implementation of clear communication with children of parents with a mental illness, pitched at an age-appropriate level, can bring a sense of relief and mitigate feelings of blame and facilitate a mutually supportive environment [19, 20]. Conversely, an absence of communication may lead to stigmatisation and an implicit belief that mental health disorders are shameful and should be hidden [15, 21, 22].

Very little research has been done on communicating with children of parents with a mental illness in a low-resource African context. People with mental illness in South Africa often report feeling stigmatised about their illness [23], which can negatively impact on communication within families and social support networks [18]. Socio-cultural factors contribute to the stigma around mental illness in Africa such as the beliefs about witchcraft or influence from ancestors being the cause of the illness [7, 24]. These traditional explanatory models of mental health mean people often rely on traditional healers to cure mental illness. This reliance also reflects the accessibility, and cultural and social acceptability of this type of care [25], which is further reinforced by the scarcity and cost of biomedical alternatives. Although some traditional healers do not always share the same beliefs about the treatment of mental illness, they do report a willingness to collaborate with Western medical practices [26]. Exploring the role of stigma and traditional practices in talking to children about mental illness is essential to deepen understanding and so aid in facilitating these difficult conversations.

Global evidence suggests that healthcare professionals (HCPs) may rarely have direct contact with their patients’ children [18]; consequently children's needs when a parent has a mental illness (including their desire for communication about their parent’s illness) may be overlooked [21]. Identifying patients as parents is the initial step towards family focussed practice which aims to mitigate the impact of parental illness on the child and the family. In order to achieve service and system change in embedding family focussed practice, recommendations are well documented [27]. These include workforce education and training programmes to improve HCPs' skills and confidence about the impact of mental illness on families. Promoting family communication about illness is an important element of family focused practice; evidence suggests this improves children’s understanding of parental depression and their internalising symptoms [28]. Research in South Africa evaluated the use of structured communication frameworks which aimed to help parents talk to their children about parental physical illness. The study found that these increased rates of maternal disclosure to their children and maternal engagement with their own healthcare [29]. A similar approach could potentially be applied to mental health conditions. Effective communication aims to provide children with both an opportunity to access the information and the emotional support they require [20].

Given the high rate of mental illness in South Africa, many children will be exposed to mental illness within their family. There is a paucity of literature specific to HCPs working within sub-Saharan Africa about how to discuss with parents the impact of their mental illness on their children. Exploring HCPs’ perceptions of talking about mental illness with patients and families in South Africa could help identify key obstacles and facilitators to communication. This understanding could offer guidance about the type of support that might be needed to help HCPs have sensitive conversations with their patients, and in turn, their children.

### Study aims

This study aims to: (1) explore the experiences and perceptions of HCPs about beliefs and attitudes towards mental illness in their communities, (2) explore the experiences and perceptions of HCPs around families talking with children about a parent’s mental illness, (3) investigate HCPs’ perceptions of the obstacles and facilitators to communication with children about parental mental illness and where and with who these conversations should be initiated.

## Methods

### Study design

An interpretative qualitative design using in-depth, semi-structured interviews. This study is reported following the Consolidated Criteria for Reporting Qualitative Research (COREQ) guidelines [30].

### Setting

This study was embedded within the MRC/Wits-Agincourt Unit’s Health and Socio-Demographic Surveillance System (HSDSS), a large demographic surveillance site within Bushbuckridge, a rural, densely populated sub-district on the border with Mozambique. The site serves as the scientific foundation for a programme of advanced research and intervention studies. The majority of the population are Xitsonga speaking South Africans and a third are Mozambican refugees or their descendants. Within the study site there are 7 government-run primary healthcare clinics and 1 community health centre. These are both nurse-led, with some input from a sessional doctor up to once a week. Three district hospitals (located between 25 and 55 km from the study site) provide services to approximately 1 million inhabitants; one of these hospitals contains an inpatient Mental Healthcare Unit staffed by a family physician, nurses with psychiatric training, an occupational therapist and registered counsellor. Within Bushbuckridge there are also 2 psychologists located at one district hospital, but no government-employed psychiatrists. As in other parts of South and sub-Saharan Africa, many patients consult traditional healers including for mental health concerns [31].

### Participants

A total of 15 HCPs participated in the study. Stratified purposive sampling was used to identify a range of healthcare workers at the primary healthcare and district hospital levels who are involved in providing mental health care to the general adult population. Individuals were considered eligible if they were aged 18 and over, and had experience working with adult patients with mental illness including depression, anxiety and psychosocial distress. Individuals were excluded based upon lack of experience working with adults affected by the above mentioned conditions. Upon completion of the interview, HCPs were asked to identify additional colleagues with experience working with adults affected by mental illness. Eligible participants were contacted by MM and BM; one person did not respond to the invitation, and none declined. Written informed consent was obtained prior to the interview.

### Data collection

Semi-structured interviews were conducted by MM and BM between December 2020 and July 2021. This method was considered most appropriate for exploring broad topics whilst also providing in-depth personal information about an individual’s experience. Interviews were conducted within a private space at the place of work of the HCPs, and only the interviewer and interviewee were present during the interview. MM is an experienced qualitative fieldworker and is Xitsonga-speaking from the study area. BM is a HCP and mental health researcher with training in qualitative methods and is English-speaking. Interviews were conducted in Xitsonga and/or English based on the preference of the interviewee. A topic guide (Table 1) was developed, informed by the research aims and objectives and the research team who have extensive experience in parental mental illness and with input from BM, a HCP who has lived and worked in rural South Africa for over 7 years. The topic guide was iteratively modified as necessary to ensure follow-up with categories in subsequent interviews. Interviews were conducted face-to-face (*n* = 15), audio-recorded and lasted between 25 and 71 min (mAvg = 51). Seven interviews were conducted in English (BM) and eight in Xitsonga (MM) which were then translated into English by MM. MM is an experienced qualitative fieldworker who is fluent in Xitsonga and English. The interviews that MM conducted in Xitsonga were translated into English and transcribed by MM, so that they could ensure the original intended meaning was maintained.

**Table 1:** Topic Guide—Mental health and illness in the community

Are mental health difficulties a problem in this community?
Where do people get help? What challenges might affect people getting help?
How do patients talk about their difficulties?
How do community members talk about mental health/illness—What language do people use to talk about mental health difficulties?
How easy or difficult is it to talk about mental health?
Talking to children about mental health/illness: Do you think that people in your community talk with children about mental health and illness within the family?
What might be the challenges and benefits of talking with children about mental health and illness?
How do you think it would make a child feel to talk about mental illness?
At what sort of age do you think children start noticing if one of their parents has a mental illness?
At what sort of age do you think it would be appropriate for adults/caregivers within a family or HCPs to have conversations with children around mental health and illness?
Are there any cultural factors that might affect how people communicate with children around mental health and illness?
Who do you think is the best person to speak to children whose parents have a mental illness?
Where do you think is the best place to have these conversations? And when?
Additional topics: The impact of COVID-19 on mental health: Has COVID-19 increased stresses for people in this community? Do people talk about this stress? If so, how?

### Data analysis

Audio-recordings were transcribed verbatim and managed using NVivo V.12. Braun and Clarke’s reflexive thematic analysis framework was used to analyse the data [32, 33]. Initially the first author [LD] read and reread the transcripts to gain a sense of each HCP’s story. To ensure rigour, credibility and trustworthiness, five additional authors [BM, ER, LJD, JRH, HB] also read the transcripts. LD manually coded the data, detailing inductive descriptive codes and identified where some of them merged into themes using mind maps. Themes were discussed and refined through critical dialogue with all authors including BM and MM who conducted the interviews. BM and MM shared a written draft of study results with participants via email and in-person. Participants were asked their view on how well the study findings reflected the key obstacles and facilitators to communication with children about a parental mental illness in this community. All participants felt that the study findings were an accurate description of the situation and so the findings were not adjusted further.

### Ethical considerations

Participants were provided with oral and written information about the study and provided oral and written consent. Participants were aware of their right to withdraw from the study, as well as the option to pause, terminate or reschedule the interview. Correspondence between the researchers [BM, MM] and participants took place via email or telephonically, and all emails containing personal information were deleted at the earliest convenience. Data protection procedures were observed and assurances of confidentially were provided. Ethical approvals were obtained from the University of the Witwatersrand Human Research Ethics Committee (HREC) (R14/49), Mpumalanga Provincial Department of Health (MP_202009_001) and The Oxford Tropical Research Ethics Committee (OxTREC) (506–20).

### Research team and reflexivity

The team consisted of 7 researchers identifying as female and 3 researchers identifying as male, from a wide range of different career and life stages. Six members of the research team (BM, LD, HB, LJD, JRH, AS) have experience of working in a clinical role with patients who have a mental illness (doctor, medical student, doctor, clinical psychologist, nurse, doctor respectively). BM and MM live and work in Bushbuckridge and had established relationships with several of the HCPs that were interviewed due to other existing and ongoing mental health research projects within the study site. ER is a senior postdoctoral researcher, MM is an experienced qualitative fieldworker, and KK and ST hold academic chairs in public and population health research and established the MRC/Wits-Agincourt Research Unit in the early 1990s. All have experience of conducting academic research with a focus on promoting effective communication about physical and mental illness with children around the globe. MM identifies as Black African; BM, KK, AS and ST are White African; and LD, ER, LJD and JRH identify as White British.

## Results

A total of 15 healthcare professionals (HCPs) were recruited to take part in this study, 12 of whom identified as female and 3 of whom identified as male. HCPs’ clinical roles and sample characteristics are included in Table 2. All HCPs were working in primary or secondary care facilities in the Bushbuckridge sub-district of Mpumalanga Province, South Africa.

**Table 2:** Characteristics of the 15 participants included in the study

Variable	N
**Sex**	
Female	12
Male	3
**Clinical role**	
Medical Doctor (Family Physician)	1
Occupational Therapist	1
Professional Nurse	9
Professional Nurse with advanced psychiatric training	2
Clinical Psychologist	1
Registered Counsellor	1
**Place of work**	
Community Health Centre	2
District Hospital	6
Rural Primary Healthcare Clinic	7

HCPs described many challenges for families, the community and HCPs around communicating with children about parental mental illness. Overall, the main obstacles identified were: language and culture including cultural explanations of illness, stigma, children’s position in society, and mental health services and staff skills. The main facilitators to communication were increasing mental health awareness amongst the broader community through social media and general psychoeducation; and HCPs’ concern for the wellbeing of children of parents with a mental illness. HCPs also expressed that the involvement of the broader family in conversations around parental mental illness would be helpful in facilitating communication with children of these patients. The data below is representative of HCPs’ experience of working with patients with mental illnesses who are parents.

### Obstacle 1: Language and culture

#### Subtheme 1: Language to describe mental illness

Participants reported that local languages were “not suitable” for explaining mental health difficulties within the community, including a “lack of language to describe mental health within (African) culture or (African) languages”. They believed words like "depression' were clinical terms only used in Western medical settings.

“People are not familiar with those words. They are talking of the same illness, calling it “Ku hlangana nhloko’’ (Meaning having confusion in the head.) In our language we just have that word. We do not differentiate. They even do not know that there is depression. That is why people do not differentiate between depression and stress. To them, they are all the same things, and they call it stress (#9)”

Several HCPs reported that patients often demonstrated a lack of understanding around the symptoms of mental illness. HCPs explained that patients “don’t usually come saying they are depressed” and that they most frequently present with somatic complaints. Some HCPs reported that they had helped their patients who were parents by explaining the difference between mental health conditions and stress. HCPs felt that it was more difficult for parents to discuss their mental health concerns with their children if the patient did not have a good understanding of mental illness in general.

#### Subtheme 2: Cultural explanation of mental illness

Within the broader community, participants reported that mental illness was often attributed to the effects of witchcraft or "wrongdoing" to ancestors, and thus these same explanations would likely be used with children. HCPs reported that patients often sought help for their illness from traditional healers or local pastors. Participants described the leaders of the community as having an “established belief system for making sense of behaviours characteristic of mental illnesses which again included beliefs of being "bewitched", or due to "wrongdoing" to ancestors.

“Our culture is causing us not to be opened. Everything in our culture belongs to traditional healers. Witchcraft is the main thing. I think information needs to reach people to know that it is not everything that has been witched” (#9).

Participants reflected that if parents were more informed about mental health this could help them better explain their diagnoses to their children.

“Education is not just for children…..it does not help to educate children about mental health, meanwhile their parents still believe in witchcraft”. (#11)

### Obstacle 2: Stigma

Most participants identified stigma as a major obstacle for talking honestly about mental illness within their communities. Several participants explained that patients’ families feared how they would be treated or viewed if the patient’s mental illness was revealed to people in their community, thus creating a “significant barrier” to accessing support or increasing communication regarding mental illness.

“This is not a topic to share openly because in our culture when you say mental illness, it means upstairs you are not okay. Mental illness is not okay. We think of witchcraft. In our culture it can be a serious offence.” (#14).

Some participants felt that stigma about mental illness may be “worse” in rural communities compared with urban environments. They suggested this was because of a smaller population, where unusual behaviour was more likely to attract attention.

“This community seems to be a lot more concerned, concerned about appearance and concerned about how their family members are viewed, as opposed to in Jo’burg where it was spoken a little bit more openly.” (#2).

Some participants described children experiencing stigma arising from their parent’s illness. This could have been the child’s own stigma towards their ill parent, or stigma through association from others in the community. Participants shared their experiences of children running away from home or being provoked by peers who had heard of their parent’s mental illness. One HCP said that children can be “mean” and may ostracize a peer if they find out that their parent has a mental health condition.

“They can say your mother is mad. I am not like you whose mother is crazy.” (#9)

### Obstacle 3: Position of children in society

One participant reported that in their local culture, it was inappropriate to engage children in conversations about “adult” topics, such as mental health. Participants reflected that the wider culture within the communities of not talking about mental health has “failed children.” They suggested that the negative beliefs held by communities about mental illness included a view that such an illness would make parents unable to adequately care for their children. HCPs felt that this may contribute to a lack of communication about mental health between parent and child.

“I think the biggest thing for children is like your parent is supposed to be the person who is kind of looking after you and protecting you from everything in the world. So as soon as that relationship in your eyes has faltered… that in itself is very distressing for children”. (#1).

Many participants expressed concerns about children’s capacity to cope with information regarding a parent’s mental illness. These included fears that children would feel overburdened with the (perceived) shame or responsibility.

“Sometimes if they are still young, you know, I think it’s difficult for them to take in that information. Maybe they can be affected at school. I think it could be too much information for them. Their performance won’t be so good” (#3)

Other participants did feel that topics about parental mental health were appropriate and should be raised to start “breaking down that barrier between adults and children”. Some HCPs suggested that discussions with children about mental health would be appropriate once they were “old enough”; most HCPs considered this would be when the child was around 12-years old. However, others felt that issues of mental health could only be discussed with children after they are married and viewed as an adult in society.

“They can say a twelve-year-old child is still young to talk about those issues, we are killing his head (meaning s/he will think a lot.) That is how they say if they do not want a child to be included. Our culture starts talking with a child when s/he is married.” (#9).

### Obstacle 4: Mental health services and staff skills

HCPs discussed the broader context of patients’ access to mental healthcare treatment. Barriers included the expense and distance for patients to travel to specialist clinics, with HCPs based in general clinics noting a recent increase in the number of people with a mental illness presenting to general health services. Participants discussed the broader cultural norms of privacy relating to family matters which made it challenging to discuss mental health with some patients. One participant noted that elder patients may find it disrespectful to be asked about their mental health by an HCP of younger age to themselves.

"Patients, like old people, they hate to disclose their problems, their information to a younger people… they will say that, “Oh my gosh this kid, this baby asking me about my family problem. Who is she?”” (#4).

Most participants believed that HCPs with specialist mental health training e.g. psychologists could talk directly with children about a parent’s illness, due to their ‘qualifications’ and knowledge about mental illness. They believed this would enable them to answer children’s questions accurately. However, participants recognised the scarcity of colleagues with such skills and consequently acknowledged that conversations directly with children did not take place. Some participants reported that the family or primary caregiver were best placed to talk with children as “they know the child best” and “love the child the most”. School staff, church leaders and pastors were also discussed by participants as people who could talk with children about a parent’s illness. However, concerns were expressed that representatives of these community organizations had insufficient knowledge about mental illness and would need specialist knowledge and training before taking on this role. HCPs also discussed where the conversation ‘should be held’, with multiple recommendations of where children may prefer, ranging from an open space in a neutral setting to a clinical environment or the home setting. Non-specialist HCPs felt they lacked sufficient training to initiate conversations with parents about their children or speak to children directly.

“It would require someone who has training and who is aware of these health issues, mental health as well. They have information to be able to provide accurate and correct information to the family and not confuse them further. It requires someone qualified to do that and better able to work with children, adults and with youth.” (#6).

Participants who were practicing as professional nurses felt that inclusion of more substantial mental health awareness in their general training was necessary.

“With us [professional nurses] we are not well trained when it comes to mentally (sic) issues.” (#10)

HCPs suggested additional training such as short courses, or workshops would help them gain confidence in discussions with parents and become familiar with guidance on delivering information to children of parents with a mental illness in an age-appropriate manner.

### Facilitators 1: Social media and the Internet

Participants identified generational differences in people’s attitudes; they felt that young people were “more open” and “comfortable” talking about mental health. Some HCPs attributed this to younger people’s exposure to social media and their ability to search for information to explain their parents’ behaviour.

“They are aware, because they can just be able to Google everything that they seek. They Google and see all the persons acting like this is because of this.” (#3)

HCPs expressed that better psychoeducation of patients and their families could facilitate improved mental health awareness amongst the broader community over time. HCPs had differing opinions on who was best placed to provide psychoeducation to patients and their families, with some suggesting all HCPs should play a role and others suggesting dedicated staff with mental healthcare training would be best placed to do this, and others suggesting community healthcare workers could be equipped to fulfil this role. One participant felt that educational interventions within the community had been successful, such as Radio Shows discussing chronic health conditions. They also mentioned educational posters displayed in schools and healthcare clinics, mental health awareness talks delivered in clinic waiting rooms and educational school visits.

“Education is the key. If we educate, we are building the society.” (#13)

### Facilitators 2: Wellbeing of children

#### Subtheme 1: Family and parental role

Participants discussed the attitudes of some parents who had shown insight into how their mental illness was affecting their children. The HCPs felt that the emotions sometimes expressed by parents about their families made them more receptive to discussions about communicating with their children.

“Fathers that have become very emotional about the fact that they are ill and now in hospital. And their families are kind of living their lives and they’re maybe missing birthdays or events at school and things like that.” (#2).

HCPs reported that by raising the topic of mental illness and its impact within the family with their patients, they could then discuss with them how to overcome the challenges that their illness could raise for their children and other family members.

“Families kind of enforce the sick role. [Conversations with families could help] just to explain that this is a functional person. They can do things by themselves. Just because they happen to be in hospital for two weeks doesn’t mean then they can’t cook a meal for the family. Like not everything has to be done for them. They can kind of become part of your family again.” (#2).

#### Subtheme 2: Future mental health

Some HCPs felt that discussing mental illness with children would lead to a society where children and future generations would be more aware of mental health problems and be able to seek help.

“We must communicate so that children must be aware of these illnesses. That can also help them for future as we do not know what will happen to them. But having information will be helpful. They will know that if you take treatment, your life will be stable again.” (#8).

Participants believed that if children were encouraged to grow-up with accurate information, it would facilitate a generation of informed adults and reduce the stigma associated with mental illnesses. If these children then became parents themselves, they would possess insight into mental health, and could effectively communicate with their own children and the community.

“I think children will learn. Those who are still young can become better people. They can make better choices about their lives.” (#15)

## Discussion

HCPs in this study believed it was important for children to be informed about a parent’s mental illness; furthermore, they showed an appreciation of the benefits of communication about mental illness for children. However, many participants felt their perspectives often conflicted with community-held beliefs about the causes of mental illness and whether mental health problems were an appropriate subject to talk about, particularly with children. Participants reported that HCPs with specialist mental health knowledge would be best placed to talk to children directly about parental mental illness, and that non-specialist healthcare workers would require additional training in order to fulfil this role. The results also highlight participants’ perception that they lacked specific skills to navigate sensitive conversations with or about children of parents with a mental illness which is consistent with research conducted in other contexts [15, 34].

Cultural norms sometimes appeared a challenge for professionals in having conversations with parents about how mental health concerns may affect their children. Mental illness is commonly attributed to witchcraft or "wrongdoing the ancestors" and thus parents frequently seek support from traditional healers or local pastors [35–37]; this may be an additional obstacle for parents when considering whether to talk with children about their symptoms and illness. Some participants in the current study reflected that their own "western medical" understanding of mental illness differed from community beliefs about witchcraft. However other studies have shown that healthcare staff may also share cultural explanations for mental illness such as witchcraft [7]. Traditional beliefs about mental illness may contribute to stigma; participants felt that this represented another barrier to promoting the benefit of discussions about parental illness within the family. The participants’ reports of stigma and the impact of stigma on families in this study are consistent with the themes of a recent integrative review of stigma for families affected by parental mental illness [38]. Whilst the studies in the integrative review were mainly from North America, Europe and Australia, the study presented here is the first to include primary data from sub Saharan Africa. The fear of stigmatisation may explain why parents limit communication about their mental illness with children [15, 39]. Furthermore, parents may not tell HCPs that they have children; reasons for non-disclosure by parents include the fear of losing custody of their child(ren) or increasing their family's vulnerability to stigma [40, 41]. If HCPs are unaware that their patient is a parent, this precludes the opportunity for HCPs to share a rationale with patients about discussing their mental illness with children.

Participants described an absence of adequate professional training around addressing mental health issues (particularly for those working in primary healthcare clinics). Although guidelines around the diagnosis and treatment of mental illness are available [42, 43], these do not include consideration of traditional beliefs about mental illness or tackle the potential concerns that healthcare staff may have about such illnesses [7]. HCPs’ perceived lack of knowledge and skills around mental illness is likely to impede their confidence to raise the issue of children with their patients, or indeed offer parents a rationale for communication with children and concrete advice about how to do this. This is consistent with previous research in Europe [34, 44, 45]. The development of a step-by-step guide for HCPs may provide a helpful framework to structure these potentially sensitive conversations with patients around disclosure of their illness to children [18, 46]. However, it is vital to acknowledge that personal and professional capacity development of knowledge and skills about family-focused practice is unlikely to be translated into meaningful, sustainable progress without broader systemic changes [44, 47]. The success of such initiatives would require organisational commitments such as specific policies, resource allocation and support from healthcare management [48].

Participants identified a need to address mental health awareness more broadly within the community as an important step to increasing communication with children of parents with a mental illness. HCPs believed that psychoeducation, awareness campaigns and social media were effective at increasing the broader community’s understanding of mental illness. In Ghana and Kenya psychoeducation has already been found to effectively reduce stigma about mental illness [49]. A previous study has shown that some parents with mental illnesses express limited insight into the impact of their illness upon their children [50]. Ensuring that parents have a clear understanding of their illness, as well as the importance of communication with children, is key. Parents can then make an informed decision as to whether they discuss their mental illness with children. General public-awareness campaigns about mental illness and the possible impact on children may be particularly important given that 75% of people with a mental illness do not seek help from mental health services [6].

### Strengths and Limitations

This study addresses a gap in the literature regarding the barriers and facilitators to communication with children of parents with a mental illness in a low-resource African context. A range of participants working in both peri-urban and rural settings within the Bushbuckridge subdistrict of Mpumalanga province in South Africa were included. Interviews were conducted by interviewers who were familiar with the local culture and health system constraints. Furthermore, participants were able to conduct interviews in Xitsonga (the local language) or English according to their preference. Important contextual differences may however limit the transferability of the study findings to other African contexts.

Further work is required to understand the beliefs and attitudes about communication with children regarding mental illness amongst community members (e.g. religious leaders, educators, general public) who do not have a professional role in healthcare. The perspectives and role of community leaders (including faith and traditional healers) regarding communication with children must be explored to inform an appropriate strategy to facilitate communication with children. This must also include the opinions of parents and children with experience of parental mental illness about what is needed to support their families. Collaboration between the dual belief systems may be an effective way to support communication with children about mental health.

## Conclusions

Future research should focus on the collaborative creation of culturally sensitive evidence-based guidelines to assist HCPs in facilitating conversations with patients who are parents, and their children. Until further guidelines are available, we recommend that HCPs should be provided with materials to develop their knowledge (and thus perceived skills) about mental illness. In turn, this would enable HCPs to provide their patients with psychoeducation, enquire whether their adult patients have children, and to have discussions with their patients about the benefits of communicating with their children about the illness. Furthermore, the facilitators identified in this study could be leveraged to engage communities in discussions about mental illness, which may be useful in targeting mental health awareness and addressing the stigma which often appears to inhibit communication. Guidelines need to be supported by training for HCPs working in both primary and district healthcare facilities, as well as organisational commitments, to ensure the needs of patients’ children are not overlooked.

## Data Availability

Data are available on reasonable request. The data are deidentified participant data. Requests can be submitted to the corresponding author.

## Abbreviations

HCP: Healthcare professional
LMICs: Low and middle income countries
